# Cervical Multifidus Fatty Degeneration and Bony Foraminal Stenosis Are Associated with Unsuccessful Response to Stellate Ganglion Block in Cervical Radicular Pain: A Retrospective Study

**DOI:** 10.3390/medicina62061097

**Published:** 2026-06-05

**Authors:** Hyun-Jung Kwon, Daeyun Choi, Shin Wook Yi, Jun-Seok Lee, Jin-Woo Shin, Seong-Soo Choi, Doo-Hwan Kim, Chan-Sik Kim

**Affiliations:** Department of Anesthesiology and Pain Medicine, Asan Medical Center, University of Ulsan College, 88, Olympic-ro 43-gil, Songpa-gu, Seoul 05505, Republic of Korea; kwonhj@amc.seoul.kr (H.-J.K.);

**Keywords:** hyperostosis, nerve block, pain management, paraspinal muscles, risk factors, stellate ganglion, ultrasonography, interventional

## Abstract

*Background and Objectives*: Ultrasound-guided stellate ganglion block is a promising alternative intervention for cervical radicular pain. However, factors associated with treatment responses remain unknown. This single-center, retrospective study aimed to identify clinical and radiological factors associated with treatment response to stellate ganglion block in patients with cervical radicular pain. *Materials and Methods*: This study included patients with cervical radicular pain who underwent ultrasound-guided stellate ganglion block. Successful response was defined as a ≥4-point or ≥50% reduction in the numerical rating scale score at 3 months post-procedure compared with the pre-procedural baseline. Patients who did not meet these criteria or underwent additional interventional procedures for persistent cervical radicular pain within 3 months were classified as unsuccessful responders. Logistic regression analysis was performed to identify factors associated with treatment response. *Results*: Multivariable logistic regression analysis demonstrated that substantial cervical multifidus fatty degeneration, defined as Goutallier grade ≥2 of the cervical multifidus muscles (vs. minimal fatty degeneration, odds ratio (OR) 0.23, 95% confidence interval (CI): 0.08–0.66, *p* = 0.007) and bony hypertrophy at the uncovertebral or facet joint as the primary etiology of foraminal stenosis (vs. disc herniation, OR 0.35, 95% CI: 0.14–0.87, *p* = 0.024) were independently associated with lower odds of a successful response to stellate ganglion block. Steroid use was not significantly associated with treatment response (OR 0.52, 95% CI: 0.18–1.49, *p* = 0.223). The successful response rate declined progressively with the number of risk factors: 94.7% (18/19), 50.0% (22/44), and 40.7% (11/27) for zero, one, and two factors, respectively (*p* < 0.001). *Conclusions*: Substantial cervical multifidus fatty degeneration and foraminal stenosis attributable to bony hypertrophy may be associated with lower odds of a successful response to stellate ganglion block in patients with cervical radicular pain. These factors require prospective validation before routine clinical implementation.

## 1. Introduction

Cervical radicular pain is characterized by pain that radiates into the upper limb, typically caused by irritation or compression of a cervical nerve root due to intervertebral disc herniation or degenerative spondylotic changes that reduce neuroforaminal dimensions [1]. When conservative treatments, including exercise therapy and medication, fail to alleviate cervical radicular pain, interventional management may be considered. Cervical epidural steroid injection is one of the most widely used interventional treatments and has demonstrated efficacy in managing cervical radicular pain [2]. However, catastrophic complications such as spinal cord injury, inadvertent intrathecal injection, and anterior spinal artery syndrome can occur [3]. Furthermore, because the procedure requires fluoroscopic guidance, it carries an additional risk of radiation exposure, which can cause damage to the eyes, skin, and reproductive organs [2].

The stellate ganglion block (SGB) has emerged as a promising alternative to cervical epidural steroid injection, although its use for cervical radicular pain has not yet been fully established. The stellate ganglion provides sympathetic innervation to the head, neck, and upper extremities and is a therapeutic target for sympathetically maintained pain conditions [4]. Its blockade is believed to exert therapeutic effects by modulating sympathetic overactivity, increasing regional blood flow, and attenuating neurogenic inflammation by suppressing norepinephrine and nerve growth factor release [5,6]. Given that the pathophysiology of radicular pain involves vascular insufficiency, venous congestion, and inflammation, these mechanisms of SGB may contribute to its therapeutic effect in cervical radicular pain [7]. Consistent with these mechanisms, recent clinical evidence has shown that SGB is effective in managing cervical radicular pain, with analgesic efficacy comparable to that of cervical interlaminar epidural steroid injection [8]. Moreover, SGB can be performed under ultrasound guidance without radiation exposure and with a lower risk of catastrophic complications, offering practical advantages over epidural injection [4,8].

To date, no studies have investigated the prognostic factors associated with the treatment response to SGB. To prevent unnecessary expenses and harm to the patient, proper patient selection for SGB is needed. Therefore, the present study aimed to identify the clinical and radiological factors associated with treatment response to SGB in patients with cervical radicular pain.

## 2. Materials and Methods

### 2.1. Patients

This retrospective study was performed at the pain clinic of a tertiary referral center, in accordance with the tenets of the Declaration of Helsinki, and the study protocol was approved by the Institutional Review Board of Asan Medical Center (protocol code 2024-1179; date of approval: 8 October 2024). This study was reported in accordance with the Strengthening the Reporting of Observational Studies in Epidemiology (STROBE) guideline for observational studies. No patients or members of the public were involved in the study design and conduct, dissemination, or results and evaluation. Patient consent was waived due to the retrospective nature of the study. We reviewed the electronic medical records of consecutive patients who underwent ultrasound-guided SGB for cervical radicular pain at the pain clinic of Asan Medical Center between October 2018 and April 2023. To minimize selection bias, we screened all consecutive eligible cases during the study period using pre-specified inclusion and exclusion criteria, and we report the reasons for exclusion in a patient flow diagram. Baseline characteristics of included and excluded patients are compared in Supplementary Table S1.

The inclusion criteria were as follows: (1) unilateral cervical radicular pain; (2) evidence of cervical foraminal stenosis on magnetic resonance imaging (MRI) consistent with radicular pain; (3) age ≥ 19 years; and (4) symptoms unresponsive to at least 1 month of conservative treatment, including medication and physical therapy.

The exclusion criteria were as follows: (1) a history of cervical spine surgery; (2) bilateral symptoms or involvement of three or more segments; (3) neck pain intensity greater than arm-pain intensity; (4) other conditions, such as facet joint syndrome or myofascial pain syndrome, contributing to the symptoms; (5) cervical interventional procedures (e.g., epidural steroid injection) within 3 months prior to SGB; and (6) insufficient documentation in the electronic medical records.

### 2.2. Ultrasound-Guided Stellate Ganglion Block (SGB)

Each patient received a single ultrasound-guided SGB. All SGB procedures were performed by board-certified pain physicians, each with at least five years of experience in ultrasound-guided cervical interventional procedures. The procedure was performed with the patients in the supine position, with slight neck extension and the head rotated 5–10 degrees to the contralateral side. Monitoring equipment, including electrocardiography, a non-invasive blood pressure monitor, and a pulse oximeter, was used, and a sterile drape was applied. A high-frequency linear transducer (Samsung Medison, Seoul, Republic of Korea) was used.

The procedure was performed using a standardized approach as previously described [8]. The ultrasound probe was positioned approximately 1 cm inferior to the cricoid cartilage on the lateral aspect of the neck. The C6 root and anterior tubercle of the C6 transverse process were identified. A pre-scan of the surrounding area was conducted to ensure that the vertebral artery entered the transverse foramen at the C7 level and to check for anatomical variations in the blood vessels or nerves along the intended path of the needle. Color Doppler was used to confirm the absence of hazardous structures.

Under real-time ultrasound guidance, the final needle position was targeted at the plane between the lateral portion of the longus colli muscle and the prevertebral fascia, without making contact with the bone [9]. After confirming the proper needle position and performing aspiration to exclude intravascular placement, 6 mL of either a mixture of 1% lidocaine with steroid (dexamethasone 5 mg) or 1% lidocaine alone was injected [10]. The choice between 1% lidocaine alone and 1% lidocaine combined with dexamethasone 5 mg was based on the attending physician’s clinical judgment and the institutional practice pattern during the study period, both regimens being considered acceptable standard practice.

### 2.3. Data Collection and Outcome Measures

Pain intensity was assessed by patient self-report using an 11-point numerical rating scale (NRS, 0 = no pain, 10 = worst possible pain) at baseline and at 3 months post-SGB. The 3-month NRS was obtained at the routinely scheduled in-person follow-up visit; for patients who did not attend the in-person visit, the NRS recorded in the electronic medical record from a telephone follow-up was used. A successful response was defined a priori as either a ≥4-point or a ≥50% reduction in NRS at 3 months, in line with the IMMPACT recommendations [11]. Patients who did not meet these criteria or who underwent additional interventional procedures due to persistent cervical radicular pain within 3 months were classified as unsuccessful responders.

**Figure 1:**
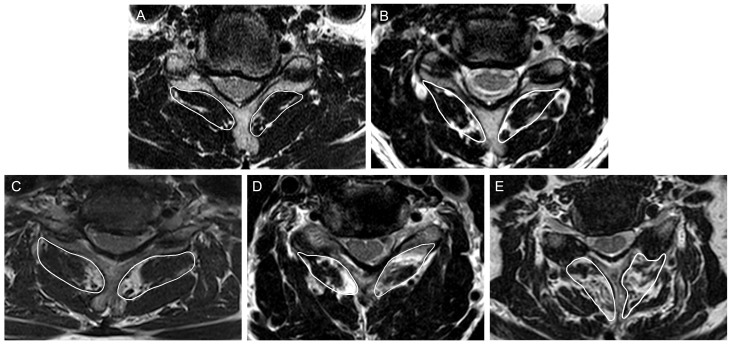
Assessment of cervical multifidus fatty degeneration using the Goutallier classification. Fatty infiltration in the bilateral multifidus muscles was evaluated using the Goutallier classification at the C5–C6 level. Axial cuts of T2-weighted magnetic resonance imaging sequences were used to assess the Goutallier classification. Each grade of the Goutallier classification was defined as follows: (**A**) Goutallier 0, no visible fat streaks; (**B**) Goutallier 1, minimal fatty streaks; (**C**) Goutallier 2, more muscle than fat; (**D**) Goutallier 3, fat and muscle present in equal quantities; and (**E**) Goutallier 4, more fat than muscle. Reproduced from Kwon, H.J.; Kim, C.S.; Kim, S.; Yoon, S.H.; Koh, J.; Kim, Y.K.; Choi, S.S.; Shin, J.W.; Kim, D.H. Association between fatty infiltration in the cervical multifidus and treatment response following cervical interlaminar epidural steroid injection. *Korean J. Pain* **2023**, 36, 358–368 [18], with permission of *The Korean Journal of Pain*.

The collected data included patient demographics, such as age, sex, body mass index (BMI), and comorbidities, including diabetes and hypertension, pre-procedural symptoms such as radicular pain with or without neck pain, neck disability index, pain duration, and procedural factors including steroid use during SGB. Cervical curvature types (lordosis, straight, sigmoidal, or kyphosis) were assessed using lateral cervical radiography before the procedure [12]. Foraminal stenosis was classified into the following three grades by comparing the narrowest foraminal width with the extraforaminal nerve root width on pre-procedural MRI: Grade 0 (normal), the foraminal width exceeded the nerve root width; Grade 1 (non-severe), the foraminal width was equal to or narrower than the nerve root width but still more than half of it; and Grade 2 (severe), the foraminal width was half or less than the nerve root width [13]. The grade of foraminal stenosis corresponding to the side of radicular pain was evaluated. Additionally, the primary etiology of foraminal stenosis was determined by differentiating between disc herniation and bony hypertrophy at the uncovertebral or facet joint. Because disc herniation and bony hypertrophy are often difficult to distinguish reliably on MRI, the etiology was instead classified on cervical oblique radiographs using a bony-priority rule: when bony foraminal encroachment attributable to uncovertebral or facet joint hypertrophy was evident on the oblique radiograph, the etiology was classified as bony hypertrophy; otherwise, it was classified as disc herniation. At the same level as foraminal stenosis, the severity of central stenosis and disc degeneration were assessed. Central stenosis was classified into the following four grades: Grade 0, normal; Grade 1, loss of >50% of the subarachnoid space without spinal cord deformation; Grade 2, spinal cord deformation without signal change; and Grade 3, increased spinal cord signal at the compressed site on T2-weighted images [14]. Disc degeneration was categorized into the following three grades: Grade 1 indicated low-intensity changes or structural alterations of the nucleus pulposus; Grade 2 referred to disc bulge or herniation accompanied by degeneration of the annulus fibrosus; and Grade 3 represented advanced degeneration with a reduction in disc height exceeding 25% [15]. Cervical multifidus fatty degeneration was evaluated at the C5–C6 level, which carries the highest biomechanical load and flexibility in the cervical spine and is therefore widely used as a reliable marker of global cervical paraspinal muscle integrity [16,17,18]. Assessing a single, anatomically consistent level also minimizes measurement variability across patients and removes the need for level-by-level adjudication when the symptomatic level differs between subjects. T2-weighted axial MRI images were analyzed, and fatty infiltration of the cervical multifidus muscle was graded using the Goutallier classification as follows: grade 0 (no fat signal), grade 1 (minimal fat signal), grade 2 (more muscle than fat), grade 3 (equal amounts of fat and muscle), and grade 4 (more fat than muscle) (Figure 1) [18,19]. For the analysis, cervical multifidus fatty degeneration was defined as minimal (Goutallier grades 0–1) or substantial (Goutallier grades 2–4) [20,21,22,23].

Three investigators (H-JK, C-SK, and S-SC) participated in the radiological assessment. All three investigators were blinded to clinical outcomes throughout the radiological evaluation. All investigators were pain physicians with over 5 years of experience interpreting spine MRI for the diagnosis of spinal pathologies. To ensure consistency in the assessment of cervical multifidus fatty degeneration, all investigators underwent training in evaluating cervical multifidus fatty degeneration using the Goutallier classification, reviewing 30 training cases each, prior to the study. Two independent investigators (H-JK and C-SK) initially evaluated the images for each parameter. If their assessments were concordant, the results were recorded in the study’s database. In cases of disagreement, a third investigator (S-SC) reviewed the images and provided the final decision. Interobserver agreement between the two primary raters was assessed using Cohen’s (unweighted) kappa (κ) for binary or nominal variables and quadratically weighted kappa for ordinal grading systems with more than two categories, with 95% confidence intervals. The percentage of cases requiring adjudication by the third investigator was also recorded.

### 2.4. Statistical Analysis

For demographic characteristics, continuous variables were summarized as mean with standard deviation or median with interquartile range, and categorical variables were expressed as absolute counts and percentages. Comparisons of demographic characteristics between the successful responder and unsuccessful responder groups were conducted using Student’s *t*-test or the Mann–Whitney U-test for continuous variables and the chi-square test or Fisher’s exact test for categorical variables, as appropriate. A complete case analysis was performed, excluding patients with incomplete clinical data from the analysis. In addition to the primary complete-case analysis, a sensitivity analysis using non-responder imputation (NRI) was performed, in which patients with missing 3-month NRS data were re-included and classified as unsuccessful responders. Univariable and multivariable logistic regression analyses were conducted to identify the factors associated with treatment response to SGB. In all logistic regression models, successful response was modeled as the outcome event; accordingly, an odds ratio of less than 1 indicates lower odds of a successful response (i.e., a higher likelihood of an unsuccessful response), whereas an odds ratio greater than 1 indicates higher odds of a successful response. The inclusion of variables in the logistic regression analysis was based on biological plausibility (including age and sex), clinical importance, and statistical considerations (*p* < 0.05 in univariable logistic regression). Variables with multicollinearity (VIF > 10) were excluded. The assumptions of the logistic regression model were assessed, including the linearity of the logit for continuous explanatory variables. Statistical significance was set at *p* < 0.05. All statistical analyses were performed using R (version 4.5.3).

## 3. Results

**Figure 2:**
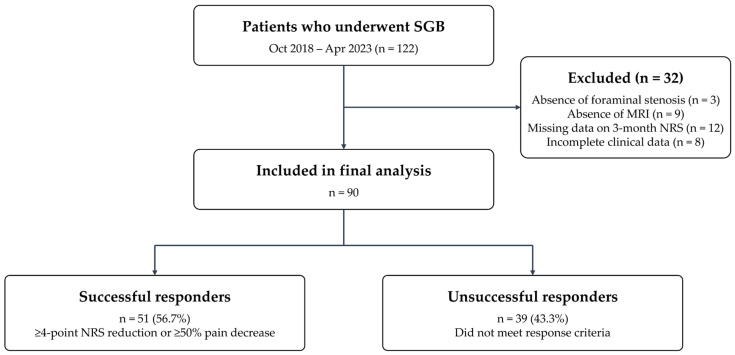
Patient flow diagram of the study. Of 122 patients assessed for eligibility, 32 were excluded (9 with an unavailable MRI, 3 with absence of foraminal stenosis on MRI, 12 with missing 3-month NRS data, and 8 with incomplete clinical data), leaving 90 patients (51 successful responders and 39 unsuccessful responders) in the final analysis.

A patient flow diagram is presented in Figure 2. Of the 122 patients who underwent SGB for cervical radicular pain during the study period, 32 were excluded—9 because of an unavailable MRI, 3 because foraminal stenosis was absent on MRI, 12 because of missing 3-month NRS data, and 8 because of incomplete clinical data—leaving 90 patients in the final analysis (51 successful responders and 39 unsuccessful responders). To assess potential selection bias, baseline characteristics of the included and excluded patients are compared on an available-case basis in Supplementary Table S1. No statistically significant differences were observed for the variables that could be compared.

The demographic characteristics of the study participants are presented in Table 1. The proportion of substantial cervical multifidus fatty degeneration was significantly higher in the unsuccessful responder group (unsuccessful responder, 76.9%; successful responder, 49.0%; *p* = 0.013). In the overall cohort, the baseline and 3-month NRS, the absolute and percentage reductions in NRS, and the proportions of patients achieving ≥2-point, ≥30%, ≥50%, and ≥4-point improvement are reported in Supplementary Table S2. Regarding the primary etiology of foraminal stenosis, which was classified into disc herniation and bony hypertrophy, the proportion of bony hypertrophy was significantly higher in the unsuccessful responder group compared with the successful responder group (unsuccessful responder, 61.5%; successful responder, 37.3%; *p* = 0.038). No other baseline characteristics differed significantly between groups (*p* > 0.05). Interobserver agreement was substantial to almost perfect across all radiological variables; the variable-specific kappa values and the corresponding proportions of cases requiring adjudication by the third investigator are presented in Supplementary Table S4.

**Figure 3:**
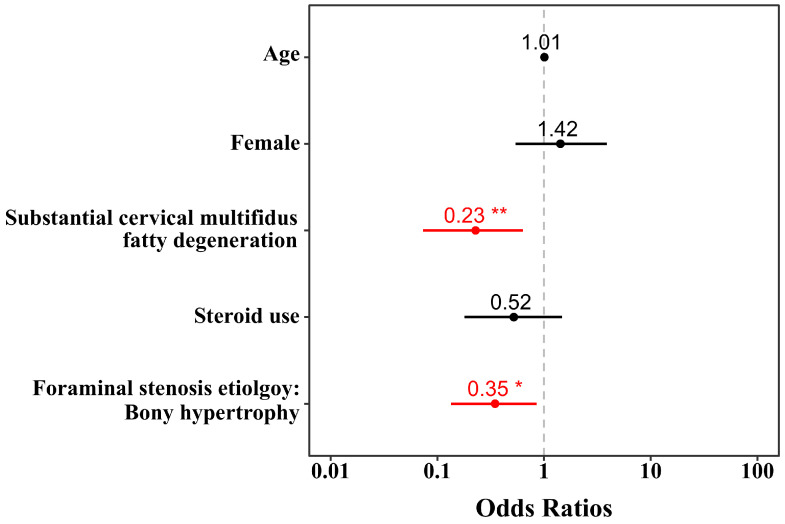
Forest plot of odds ratios with 95% confidence intervals for factors associated with treatment response in the multivariable logistic regression analysis. The dots and numbers represent actual odds ratios, and the bars denote 95% confidence intervals. Substantial cervical multifidus fatty degeneration (Goutallier grade ≥ 2) and bony hypertrophy as the primary etiology of foraminal stenosis were factors independently associated with lower odds of a successful response to ultrasound-guided stellate ganglion block. * *p* < 0.05, ** *p* < 0.01.

The results of logistic regression analysis are presented in Table 2 and Figure 3; among all evaluated variables, two variables emerged as factors independently associated with lower odds of a successful response. Multivariable logistic regression analysis revealed that substantial multifidus fatty degeneration (vs. minimal multifidus fatty degeneration, odds ratio (OR) 0.23, 95% confidence interval (CI): 0.08–0.66, *p* = 0.007) and bony hypertrophy as the primary etiology of foraminal stenosis (vs. disc herniation, OR 0.35, 95% CI: 0.14–0.87, *p* = 0.024) were independently associated with lower odds of a successful response at 3 months post-SGB. In contrast, steroid use had no significant association with treatment response (OR 0.52, 95% CI: 0.18–1.49, *p* = 0.223). All variables in the final multivariable model had VIFs below 2, indicating no meaningful multicollinearity. In the NRI sensitivity analysis, substantial multifidus fatty degeneration and bony hypertrophic foraminal stenosis remained independently associated with lower odds of a successful response; the corresponding adjusted odds ratios with 95% confidence intervals are reported in Supplementary Table S3.

**Figure 4:**
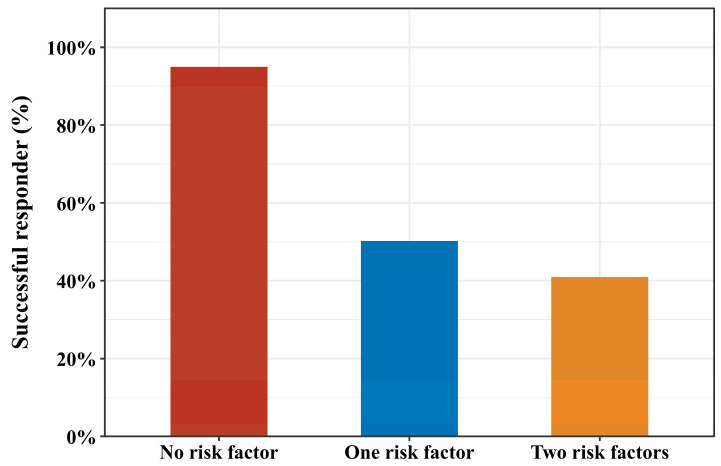
Risk factors are defined as (i) substantial cervical multifidus fatty degeneration (Goutallier grade ≥ 2) and (ii) foraminal stenosis predominantly attributable to bony hypertrophy at the uncovertebral or facet joint. The bars show the successful-response rate at 3 months after stellate ganglion block in patients with 0, 1, and 2 of these risk factors.

The successful response rate was 44.2% (19/43) among patients with bony hypertrophic foraminal stenosis, and 45.5% (25/55) among those with substantial cervical multifidus fatty degeneration. The successful-response rate differed across groups defined by the number of risk factors: 94.7% (18/19) in patients with no risk factor, 50.0% (22/44) in patients with one risk factor, and 40.7% (11/27) in patients with two risk factors (*p* < 0.001; Figure 4), corresponding to an absolute difference of more than 50 percentage points between patients with no and two risk factors.

## 4. Discussion

This retrospective study showed that substantial cervical multifidus fatty degeneration and bony hypertrophy as the primary etiology of foraminal stenosis were independently associated with lower odds of a successful response to SGB, with both factors reducing the odds of a successful response by approximately 65–77% compared with their respective reference categories. Notably, the successful-response rate declined progressively with the number of risk factors, from 94.7% in patients with no risk factor to 50.0% and 40.7% in those with one and two risk factors, respectively. Steroid use showed no significant association with treatment response.

This study identified substantial cervical multifidus fatty degeneration, defined as fatty infiltration of the multifidus muscles with a Goutallier grade ≥2, as a negative prognostic factor for response to SGB. The multifidus muscle plays a critical role in spinal stability, proprioception, and postural control [24,25,26]. Owing to its high proportion of type I oxidative fibers, the multifidus muscle is particularly vulnerable to degeneration under conditions of chronic disuse, denervation, and inflammation [27,28]. Its degeneration may further exacerbate spinal biomechanical stress and contribute to a self-perpetuating cycle of pain and muscle atrophy [26]. In this context, one possible explanation is that multifidus fatty degeneration may be associated with central sensitization and reduced pain thresholds. Moreover, fatty infiltration—considered a hallmark of chronic myopathic transformation—has been correlated with poor clinical outcomes in spinal disorders [28]. The Goutallier classification provides a reproducible and clinically applicable method for grading fatty infiltration in muscles [29,30]; prior studies have shown that a Goutallier grade ≥2 is associated with diminished treatment response across multiple spinal interventions [18,31,32,33]. The mechanisms linking fatty infiltration of the multifidus muscles to poor SGB outcomes may involve both neural and autonomic factors. Chronic nerve root compression can lead to combined somatic and sympathetic denervation, which may stimulate adipogenic processes and further muscle degeneration [27]. Given that SGB primarily targets the sympathetic nervous system, it is conceivable that pre-existing autonomic dysfunction related to chronic sympathetic denervation could limit its therapeutic efficacy. Furthermore, muscle disuse and deconditioning, reflected by fatty infiltration, have been associated with heightened central sensitization and poor treatment responsiveness, providing another possible explanation for the observed findings [34]. Importantly, because this study was retrospective and observational and did not include direct neurophysiological, inflammatory, or biomechanical measurements, these mechanistic interpretations should be regarded as hypothesis-generating rather than definitive and warrant confirmation in dedicated mechanistic studies. Moreover, multifidus fatty degeneration may reflect a broader process of chronicity, inactivity, or generalized degeneration rather than a direct causal driver of the lower odds of a successful response to SGB [35]; we therefore interpret it as a clinically accessible imaging marker that may flag patients with a less favorable prognosis after SGB.

Beyond multifidus fatty degeneration, this study also found that cervical foraminal stenosis attributable to prominent bony hypertrophy was associated with lower odds of a successful response to SGB. Foraminal nerve root compression can result from bony hypertrophy, disc herniation, or a combination of both [36]. Previous studies have shown that patients with disc herniation achieved better outcomes with treatments such as cervical epidural steroid injections, compared with those with spondylosis-related foraminal stenosis [37,38]. Disc herniation may represent a more reversible etiology because it is associated with chemical inflammatory reactions and potential resorption of disc material, possibly contributing to more favorable outcomes. In contrast, bony hypertrophic stenosis is primarily related to fixed mechanical nerve root compression, which is generally considered irreversible [37]. This pathophysiological difference may explain why prominent bony hypertrophy was identified as a factor associated with lower odds of a successful response after SGB in the present study. In addition, the radiological severity of foraminal stenosis was not associated with treatment response in the present study, consistent with previous reports that the radiological severity does not necessarily correlate with clinical symptoms [39,40].

Regarding the role of steroids, previous studies on cervical epidural injections have generally shown limited additional benefits beyond local anesthetics, except in cases of disc herniation [2]. Although steroids are known to inhibit phospholipase A_2_, suppress C-fiber activity, and reduce capillary permeability, their clinical efficacy appears to be limited, with local anesthetics thought to be the primary active agents [2,41]. Given that even perineural steroid injections show uncertain effectiveness, it is unlikely that steroid administration around sympathetic nerves, which are anatomically distant from the lesion, would yield meaningful benefits. This was supported by a recent study demonstrating that SGB with local anesthetic alone was non-inferior to cervical epidural injection using both steroids and local anesthetics, suggesting that steroids are not essential for clinical improvement [8]. In the current study, steroid use was not associated with treatment response post-SGB. Although corticosteroids have well-established anti-inflammatory and neuromodulatory effects, local anesthetics are generally regarded as the primary therapeutic agents for SGB [42].

Clinically, our findings may help inform patient selection and counseling rather than serve as a firm decision rule. In patients with substantial cervical multifidus fatty degeneration and/or foraminal stenosis predominantly attributable to bony hypertrophy, clinicians may explicitly discuss the lower probability of a favorable response to SGB and consider alternative interventions. However, imaging findings alone should not be used to deny SGB to symptomatic patients, particularly given the favorable safety profile of ultrasound-guided SGB. Prospective, multicenter validation studies—including assessment of model calibration and external generalizability—are required before these MRI-based factors can be incorporated into routine clinical decision-making.

This study had some limitations. First, cervical multifidus fatty degeneration was assessed based on fatty infiltration of the multifidus muscles at a single C5–C6 level. However, this method has been widely adopted in previous studies and has shown significant associations with clinical outcomes [18,20,21]; therefore, it can be considered a reliable indicator of the overall condition of the cervical muscles. Second, this study was conducted at a single center with participants exclusively of East Asian origin, which may limit the generalizability of the findings. Further investigations are needed to confirm the external validity. Third, the retrospective design inherently restricts control over confounding variables, and residual confounding from unmeasured variables including psychological status, pain-related catastrophizing, central sensitization indices, and occupational and physical activity cannot be excluded. To address this limitation, we applied various statistical methods, including multivariable logistic regression analysis, which may have contributed to reducing confounding effects and thereby enhancing the reliability of the results. Fourth, because the present study was retrospective, the medication of the study participants was not controlled, and analgesic exposure was typically shared across multiple prescribers and over-the-counter sources; owing to this limitation, medication use was not evaluated. In addition, assessment of psychiatric or psychological comorbidities was not routinely performed at our institution, and these factors could not be incorporated into the analysis. Both are important sources of residual confounding and should be addressed in future prospective studies. Fifth, despite pre-specified eligibility criteria, selection bias due to complete-case analysis and indication bias inherent to retrospective procedural studies remain possible; a non-responder imputation sensitivity analysis, in which patients with missing 3-month NRS data were re-included and classified as unsuccessful responders, yielded consistent findings. Sixth, treatment response was assessed solely on the basis of pain intensity (NRS), as data on the other core outcome domains recommended for chronic pain trials—such as the neck disability index—were unavailable, and follow-up was limited to 3 months, precluding assessment of long-term durability. Future studies should additionally evaluate these domains, including physical functioning, emotional functioning, and patient-rated global improvement, with long-term follow-up. Seventh, although the classification of additional interventional procedures due to persistent cervical radicular pain within 3 months as an unsuccessful response was prespecified in the outcome definition, it nonetheless relied in part on physician judgment and may not generalize to other practice settings. Eighth, the modest sample size and the events-per-variable ratio mean that the multivariable model may be subject to overfitting; we did not perform external validation, and prospective multicenter studies are needed to confirm our findings. Ninth, mechanistic interpretations should be regarded as hypothesis-generating, as no direct neurophysiological, inflammatory, or biomechanical measures were obtained. Finally, given the observational nature of the study, it was limited to identifying associations rather than establishing causal relationships between independent and dependent variables.

## 5. Conclusions

In conclusion, substantial cervical multifidus fatty degeneration and foraminal stenosis attributable to bony hypertrophy were independently associated with lower odds of a successful response at 3 months post-SGB in patients with cervical radicular pain. Pre-procedural MRI-based assessment of cervical multifidus fatty degeneration and foraminal stenosis etiology may help identify patients with a lower probability of response to SGB. However, prospective validation is required before these factors can be used for routine clinical decision-making.

## Figures and Tables

**Table 1:** Baseline demographics and clinical characteristics.

Variables	Unsuccessful Responder (*n* = 39)	Successful Responder (*n* = 51)	Total (*n* = 90)	*p* Value
Age, years	58.1 ± 10.9	56.5 ± 11.1	57.2 ± 11.0	0.483
Sex, female	14 (35.9%)	21 (41.2%)	35 (38.9%)	0.771
BMI, kg/m^2^	24.1 (22.5–26.5)	24.0 (22.1–26.1)	24.0 (22.4–26.2)	0.729
Diabetes	7 (17.9%)	8 (15.7%)	15 (16.7%)	>0.999
Hypertension	13 (33.3%)	9 (17.6%)	22 (24.4%)	0.142
Pre-procedural symptom				0.250
Radicular pain only	24 (61.5%)	24 (47.1%)	48 (53.3%)	
Radicular and neck pain	15 (38.5%)	27 (52.9%)	42 (46.7%)	
Pre-procedural NRS	5.0 (4.0–7.0)	6.0 (4.0–6.0)	5.0 (4.0–6.0)	0.553
Neck disability index	13.5 ± 6.1	13.8 ± 5.8	13.7 ± 5.9	0.821
Pain duration, months	4.0 (2.5–9.5)	3.0 (3.0–6.0)	3.0 (3.0–7.0)	0.236
Cervical curve				0.725
Lordosis	11 (28.2%)	11 (21.6%)	22 (24.4%)	
Straight	22 (56.4%)	30 (58.8%)	52 (57.8%)	
Sigmoidal or kyphosis	6 (15.4%)	10 (19.6%)	16 (17.8%)	
Central stenosis				0.653
Grade 0	2 (5.1%)	5 (9.8%)	7 (7.8%)	
Grade 1	19 (48.7%)	21 (41.2%)	40 (44.4%)	
Grade 2	18 (46.2%)	25 (49.0%)	43 (47.8%)	
Foraminal stenosis grade				0.936
Grade 1	5 (12.8%)	8 (15.7%)	13 (14.4%)	
Grade 2	34 (87.2%)	43 (84.3%)	77 (85.6%)	
Foraminal stenosis level				0.586
C4–C5	3 (7.7%)	3 (5.9%)	6 (6.7%)	
C5–C6	18 (46.2%)	29 (56.9%)	47 (52.2%)	
C6–C7	17 (43.6%)	19 (37.3%)	36 (40.0%)	
C7–C8	1 (2.6%)	0 (0.0%)	1 (1.1%)	
Primary etiology of foraminal stenosis				0.038
Disc herniation	15 (38.5%)	32 (62.7%)	47 (52.2%)	
Bony hypertrophy	24 (61.5%)	19 (37.3%)	43 (47.8%)	
Disc degeneration				0.380
Grade 1	18 (46.2%)	24 (47.1%)	42 (46.7%)	
Grade 2	12 (30.8%)	10 (19.6%)	22 (24.4%)	
Grade 3	9 (23.1%)	17 (33.3%)	26 (28.9%)	
Cervical multifidus fatty degeneration				0.013
Minimal	9 (23.1%)	26 (51.0%)	35 (38.9%)	
Substantial	30 (76.9%)	25 (49.0%)	55 (61.1%)	
Steroid use	13 (33.3%)	11 (21.6%)	24 (26.7%)	0.312

Age and neck disability index are expressed as mean ± standard deviation (SD); body mass index, pre-procedural numerical rating scale, and pain duration are expressed as median (interquartile range); and all categorical variables are expressed as number (%). BMI, body mass index; NRS, numerical rating scale.

**Table 2:** Univariable and multivariable logistic regression analyses for factors associated with successful response after stellate ganglion block.

Variables	Univariable Analysis		Multivariable Analysis	
	Odds Ratio (95% CI)	*p* Value	Odds Ratio (95% CI)	*p* Value
Age, years	0.99 (0.95–1.02)	0.478	1.01 (0.96–1.05)	0.723
Sex, female	1.25 (0.53–2.95)	0.611	1.42 (0.53–3.79)	0.480
BMI, kg/m^2^	1.00 (0.88–1.14)	0.976		
Diabetes	0.85 (0.28–2.59)	0.775		
Hypertension	0.43 (0.16–1.14)	0.090		
Pre-procedural symptom				
Radicular pain only	Reference			
Radicular and neck pain	1.80 (0.77–4.20)	0.174		
Pre-procedural NRS	1.12 (0.87–1.44)	0.393		
Neck disability index	1.01 (0.94–1.08)	0.818		
Pain duration, months	0.97 (0.92–1.01)	0.124		
Cervical curve				
Lordosis	Reference			
Straight	1.36 (0.50–3.71)	0.543		
Sigmoidal or kyphosis	1.67 (0.45–6.19)	0.446		
Foraminal stenosis grade				
Grade 1	Reference			
Grade 2	0.79 (0.24–2.64)	0.702		
Foraminal stenosis level				
C4–C5	Reference			
C5–C6	1.61 (0.29–8.86)	0.584		
C6–C7	1.12 (0.20–6.30)	0.900		
C7–C8	0.00 (0.00–Inf)	0.992		
Primary etiology of foraminal stenosis				
Disc herniation	Reference		Reference	
Bony hypertrophy	0.37 (0.16–0.88)	0.024	0.35 (0.14–0.87)	0.024
Disc degeneration				
Grade 1	Reference			
Grade 2	0.62 (0.22–1.76)	0.375		
Grade 3	1.42 (0.51–3.90)	0.500		
Central stenosis				
Grade 0	Reference			
Grade 1	0.44 (0.08–2.55)	0.362		
Grade 2	0.56 (0.10–3.19)	0.510		
Cervical multifidus fatty degeneration				
Minimal	Reference		Reference	
Substantial	0.29 (0.11–0.73)	0.008	0.23 (0.08–0.66)	0.007
Steroid use	0.55 (0.21–1.41)	0.214	0.52 (0.18–1.49)	0.223

BMI, body mass index—CI, confidence interval—NRS, numerical rating scale.

## Data Availability

The data presented in this study are available on request from the corresponding author. The data are not publicly available due to them containing information that could compromise the privacy of research participants.

## Supplementary Materials

The following supporting information can be downloaded at https://www.mdpi.com/article/10.3390/medicina62061097/s1. Supplementary Table S1: Comparison of baseline characteristics between included and excluded patients on an available-case basis; Supplementary Table S2: Continuous and threshold-based pain outcomes at 3 months after stellate ganglion block in the overall cohort; Supplementary Table S3: Non-responder imputation sensitivity analysis of factors associated with successful response after stellate ganglion block; Supplementary Table S4: Interobserver agreement (Cohen’s kappa) for the radiological assessments, including κ (or weighted κ) with 95% confidence intervals and the percentage of cases requiring adjudication.

## Abbreviations

The following abbreviations are used in this manuscript:

BMI	Body mass index
MRI	Magnetic resonance imaging
NRS	Numeric rating scale
SGB	Stellate ganglion block
