# Supplementary material for: Cervical Multifidus Fatty Degeneration and Bony Foraminal Stenosis Are Associated with Unsuccessful Response to Stellate Ganglion Block in Cervical Radicular Pain: A Retrospective Study
Source: Medicina (Kaunas). 2026 Jun 5;62(6):1097. doi: 10.3390/medicina62061097 (PMC13303820; doi:10.3390/medicina62061097)
Supplement: Supplementary file 1 [file medicina-62-01097-s001.zip › Supplementary Table S4.pdf]

Supplementary Table S4. Interobserver agreement (Cohen's kappa) for the radiological assessments

Radiological variable	Kappa type	κ (95% CI)	Observed agreement, %	Cases adjudicated, n (%)
Cervical multifidus fatty degeneration	Unweighted	0.842 (0.730–0.954)	92.2	7 (7.8)
Foraminal stenosis etiology	Unweighted	0.845 (0.737–0.954)	92.2	7 (7.8)
Foraminal stenosis grade	Unweighted	0.850 (0.685–1.000)	96.7	3 (3.3)
Central canal stenosis grade	Quadratic-weighted	0.902 (0.838–0.965)	91.1	8 (8.9)
Disc degeneration grade	Quadratic-weighted	0.928 (0.880–0.976)	90.0	9 (10.0)
Cervical curvature	Unweighted	0.850 (0.751–0.949)	91.1	8 (8.9)

Interobserver agreement was assessed between the two primary raters (H-JK and C-SK) before adjudication. Cohen's (unweighted) kappa was used for binary or nominal variables and quadratic-weighted kappa for ordinal grading systems with more than two categories; 95% confidence intervals were estimated analytically. A third investigator (S-SC) adjudicated disagreements; across the six variables, adjudication was required in 42 of 540 assessments (7.8%). CI, confidence interval.
