# Supplementary material for: Cervical Multifidus Fatty Degeneration and Bony Foraminal Stenosis Are Associated with Unsuccessful Response to Stellate Ganglion Block in Cervical Radicular Pain: A Retrospective Study
Source: Medicina (Kaunas). 2026 Jun 5;62(6):1097. doi: 10.3390/medicina62061097 (PMC13303820; doi:10.3390/medicina62061097)
Supplement: Supplementary file 1 [file medicina-62-01097-s001.zip › Supplementary Table S1.pdf]

Supplementary Table S1. Comparison of baseline characteristics between included and excluded patients on an available-case basis

Variables	Analyzed, n (Included / Excluded)	Included	Excluded	P-value
Age, years	90 / 32	57.2 ± 11.0	56.2 ± 8.9	0.641
Sex, female	90 / 32	35 (38.9%)	18 (56.2%)	0.135
BMI, kg/m^2	90 / 24	24.0 (22.4–26.2)	23.5 (21.7–25.5)	0.555
Diabetes	90 / 32	15 (16.7%)	4 (12.5%)	0.778
Hypertension	90 / 32	22 (24.4%)	12 (37.5%)	0.236
Pre-procedural symptom	90 / 19			0.913
Radicular pain only		48 (53.3%)	11 (57.9%)	
Radicular and neck pain		42 (46.7%)	8 (42.1%)	
Pre-procedural NRS	90 / 32	5.0 (4.0–6.0)	5.0 (4.0–6.0)	0.159
Neck disability index	90 / 31	13.5 (10.0–18.0)	13.0 (10.5–17.5)	0.868
Pain duration, months	90 / 20	3.0 (3.0–7.0)	3.0 (2.0–5.5)	0.556
Cervical curve	90 / 20			0.391
Lordosis		22 (24.4%)	5 (25.0%)	
Straight		52 (57.8%)	14 (70.0%)	
Sigmoidal or kyphosis		16 (17.8%)	1 (5.0%)	
Central stenosis	90 / 20			0.224
Grade 0		7 (7.8%)	0 (0.0%)	
Grade 1		40 (44.4%)	13 (65.0%)	
Grade 2		43 (47.8%)	7 (35.0%)	
Foraminal stenosis	90 / 20			0.508
Grade 1		13 (14.4%)	4 (20.0%)	
Grade 2		77 (85.6%)	16 (80.0%)	
Foraminal stenosis level	90 / 20			0.350
C4–C5		6 (6.7%)	0 (0.0%)	
C5–C6		47 (52.2%)	9 (45.0%)	
C6–C7		36 (40.0%)	10 (50.0%)	
C7–C8		1 (1.1%)	1 (5.0%)	
Primary etiology of foraminal stenosis	90 / 20			0.702
Disc herniation		47 (52.2%)	12 (60.0%)	
Bony hypertrophy		43 (47.8%)	8 (40.0%)	
Disc degeneration	90 / 20			0.258
Grade 1		42 (46.7%)	13 (65.0%)	
Grade 2		22 (24.4%)	2 (10.0%)	
Grade 3		26 (28.9%)	5 (25.0%)	
Cervical multifidus fatty	90 / 20			>0.999

Variables	Analyzed, n (Included / Excluded)	Included	Excluded	P-value
degeneration				
Minimal		35 (38.9%)	8 (40.0%)	
Substantial		55 (61.1%)	12 (60.0%)	
Steroid use	90 / 20	24 (26.7%)	8 (40.0%)	0.360

Values are expressed as mean ± SD, median (interquartile range), or number (%). "Analyzed, n" indicates the number of patients with available data for each variable in the included and excluded groups–this number differs among excluded patients because exclusion was based on incomplete data and not all excluded patients underwent radiological evaluation.

BMI, body mass index– NRS, numerical rating scale.
