# Supplementary material for: Cervical Multifidus Fatty Degeneration and Bony Foraminal Stenosis Are Associated with Unsuccessful Response to Stellate Ganglion Block in Cervical Radicular Pain: A Retrospective Study
Source: Medicina (Kaunas). 2026 Jun 5;62(6):1097. doi: 10.3390/medicina62061097 (PMC13303820; doi:10.3390/medicina62061097)
Supplement: Supplementary file 1 [file medicina-62-01097-s001.zip › Supplementary Table S2.pdf]

Supplementary Table S2. Continuous and threshold-based pain outcomes at 3 months after stellate ganglion block in the overall cohort

Outcome measure	Overall cohort (N = 90)
Baseline NRS, mean ± SD	5.4 ± 1.7
3-month NRS, mean ± SD	2.5 ± 1.8
Absolute NRS change, mean ± SD	2.9 ± 2.1
Percentage NRS change (%), mean ± SD	51.9 ± 33.9
Patients achieving ≥2-point improvement, n (%)	69 (76.7%)
Patients achieving ≥30% improvement, n (%)	66 (73.3%)
Patients achieving ≥50% improvement, n (%)	57 (63.3%)
Patients achieving ≥4-point improvement, n (%)	36 (40.0%)

NRS, numerical rating scale.
