# Supplementary material for: Cervical Multifidus Fatty Degeneration and Bony Foraminal Stenosis Are Associated with Unsuccessful Response to Stellate Ganglion Block in Cervical Radicular Pain: A Retrospective Study
Source: Medicina (Kaunas). 2026 Jun 5;62(6):1097. doi: 10.3390/medicina62061097 (PMC13303820; doi:10.3390/medicina62061097)
Supplement: Supplementary file 1 [file medicina-62-01097-s001.zip › Supplementary Table S3.pdf]

Supplementary Table S3. Non-responder imputation sensitivity analysis of factors associated with successful response after stellate ganglion block

Variables	Univariable Analysis Odds Ratio (95% CI)	Univariable Analysis P value	Multivariable Analysis Odds Ratio (95% CI)	Multivariable Analysis P value
Age, years	1.00 (0.96–1.03)	0.798	1.01 (0.97–1.06)	0.505
Sex, female	1.14 (0.51–2.54)	0.744	1.21 (0.50–2.94)	0.672
BMI, kg/m^2	1.02 (0.91–1.14)	0.767		
Diabetes	1.14 (0.38–3.43)	0.812		
Hypertension	0.46 (0.18–1.16)	0.099		
Pre-procedural symptom				
Radicular pain only	Reference			
Radicular and neck pain	1.32 (0.60–2.89)	0.486		
Pre-procedural NRS	1.08 (0.85–1.38)	0.520		
Neck disability index	1.00 (0.94–1.07)	0.984		
Pain duration, months	0.97 (0.93–1.01)	0.169		
Cervical curve				
Lordosis	Reference			
Straight	1.27 (0.50–3.25)	0.614		
Sigmoidal or kyphosis	2.12 (0.59–7.66)	0.251		
Foraminal stenosis grade				
Grade 1	Reference			
Grade 2	0.88 (0.29–2.63)	0.812		
Foraminal stenosis level				
C4–C5	Reference			
C5–C6	1.21 (0.22–6.54)	0.826		
C6–C7	0.90 (0.16–5.03)	0.909		
C7–C8	0.00 (0.00–Inf)	0.988		
Primary etiology of foraminal stenosis				
Disc herniation	Reference		Reference	
Bony hypertrophy	0.43 (0.19–0.96)	0.038	0.41 (0.18–0.96)	0.040
Disc degeneration				
Grade 1	Reference			
Grade 2	0.77 (0.28–2.09)	0.607		
Grade 3	1.31 (0.52–3.27)	0.566		
Central stenosis				
Grade 0	Reference			
Grade 1	0.32 (0.06–1.84)	0.203		
Grade 2	0.45 (0.08–2.58)	0.374		
Cervical multifidus fatty degeneration				
Minimal	Reference		Reference	
Substantial	0.34 (0.15–0.78)	0.011	0.29 (0.12–0.74)	0.009
Steroid use	0.58 (0.24–1.43)	0.239	0.53 (0.20–1.41)	0.204

The non-responder imputation sensitivity analysis included 101 patients: the 90 patients of the primary complete-case analysis plus 11 of the 12 patients with missing 3-month NRS data, who were re-included and classified as unsuccessful responders. The remaining 1 patient was excluded because of missing BMI data. BMI, body mass index– CI, confidence interval– NRS, numerical rating scale.
